# Online Doppler Effect Elimination Based on Unequal Time Interval Sampling for Wayside Acoustic Bearing Fault Detecting System

**DOI:** 10.3390/s150921075

**Published:** 2015-08-27

**Authors:** Kesai Ouyang, Siliang Lu, Shangbin Zhang, Haibin Zhang, Qingbo He, Fanrang Kong

**Affiliations:** Department of Precision Machinery and Precision Instrumentation, University of Science and Technology of China, Hefei 230026, China; E-Mails: oukesai@mail.ustc.edu.cn (K.O.); lusliang@mail.ustc.edu.cn (S.L.); zsbss@mail.ustc.edu.cn (S.Z.); zhbzhbyr@mail.ustc.edu.cn (H.Z.)

**Keywords:** acoustic signal, Doppler effect, unequal time interval sampling scheme, online fault diagnosis, embedded system, train bearings

## Abstract

The railway occupies a fairly important position in transportation due to its high speed and strong transportation capability. As a consequence, it is a key issue to guarantee continuous running and transportation safety of trains. Meanwhile, time consumption of the diagnosis procedure is of extreme importance for the detecting system. However, most of the current adopted techniques in the wayside acoustic defective bearing detector system (ADBD) are offline strategies, which means that the signal is analyzed after the sampling process. This would result in unavoidable time latency. Besides, the acquired acoustic signal would be corrupted by the Doppler effect because of high relative speed between the train and the data acquisition system (DAS). Thus, it is difficult to effectively diagnose the bearing defects immediately. In this paper, a new strategy called online Doppler effect elimination (ODEE) is proposed to remove the Doppler distortion online by the introduced unequal interval sampling scheme. The steps of proposed strategy are as follows: The essential parameters are acquired in advance. Then, the introduced unequal time interval sampling strategy is used to restore the Doppler distortion signal, and the amplitude of the signal is demodulated as well. Thus, the restored Doppler-free signal is obtained online. The proposed ODEE method has been employed in simulation analysis. Ultimately, the ODEE method is implemented in the embedded system for fault diagnosis of the train bearing. The results are in good accordance with the bearing defects, which verifies the good performance of the proposed strategy.

## 1. Introduction

Due to rapid development of the national economy and society, requirement for transportation capability has been increased considerably. As one of the most important means of transportation, the railway is extremely important because of its high speed and strong transportation capability. However, unexpected breakdowns may lead to serious accidents and damages to the train transportation system, which would result in large economic losses [1]. In this circumstance, it is urgently required to guarantee the stability, security and continuous operation of train transportation for passengers and freight. Bearing defect is one of the most dominant fault types that cause the trains’ breakdown [2]. A slight defect on the rolling bearing may force the train to stop, and a serious fault may lead to major accidents and bring about substantial damage [3]. Therefore, it is profoundly important to develop the techniques of train bearing condition monitoring and fault diagnosis [4].

There exists two common kinds of train bearing condition monitoring systems nowadays, one is the on train detection system (OTDS) and the other is the wayside acoustic defective bearing detector system (ADBD) [2,5]. Since its advent, several diagnostic strategies have been proposed for OTDS, such as oil monitoring [6], temperature detecting [7,8], vibration signal analysis [9,10,11], and acoustic emission diagnosis [12,13,14] methods. However, the oil monitoring strategy is only suitable for lubrication bearing in low velocity situations [15]. The temperature detecting method can only detect serious faults of the train bearing, as only serious faults happen in the bearing, the bearing temperature raises. That would bring about a tremendous threat to the train transportation. Meanwhile, for the acoustic emission diagnosis technique, because of signal attenuation and the difficulty in signal processing and classification, there still exist some problems for application. Moreover, for all the techniques taken in the OTDS, they all suffer from the drawbacks of value and size. Hundreds of sensors should be fabricated on the train as hundreds of bearings must be monitored and the situation of each monitored bearing is comparatively complex [16]. Besides, a set of OTDS can only monitor one train, which makes the system uneconomic.

Subsequently, when it comes to the ADBD, the acoustic signal analysis method [17,18] is included in the system as this method can detect the incipient defect of the train bearing. While comparing with the OTDS, the ADBD requires less sensors and thousands of bearings can be detected when trains pass by the system each day, as all of the hardware devices are placed by the trackside [19]. Furthermore, the diagnosis result of ADBD is more reliable than that of OTDS [3]. In addition, the ADBD can detect the incipient defect of the bearing degradation process to prevent a grave accident happening [20]. However, as the trains move at a high relative speed to the microphones that are placed by the wayside, the Doppler effect is introduced in the acquired signal [21]. Especially, frequency shift and frequency band expansion would appear in the spectrum of the acquired acoustic signal. Besides, the amplitude of the origin signal would be modulated according to the Morse acoustic theory. As a consequence, the performance of the diagnosis system would be brought down greatly, especially when the train is passing by at a high speed.

To overcome the aforementioned problem in the ADBD system, He *et al.* [22] proposed a method which is a combination of signal resampling and information enhancement. The Doppler distorted acoustic signal is first reduced by the new resampling method according to a frequency variation curve extracted from the time-frequency domain. Dybała [21] put forward a disturbance-oriented dynamic signal resampling method based on the Hilbert transform (HT) to remove the Doppler effect. Liu *et al.* [23,24] presented a time-domain interpolation resampling (TIR) method to eliminate the Doppler distortion and Shen *et al.* [25] proposed a Doppler transient model based on the Laplace wavelet and spectrum correlation assessment for the Doppler distorted signal. Nevertheless, most of the proposed strategies eliminate the Doppler distortion by processing the acquired signal off-line, including the methods aforementioned. However, the diagnosis speed is vital for the ADBD. The less time the ADBD consumed, the higher probability that grave casualty is avoided and immeasurable economic benefit is added. So, it is meaningful to investigate an online Doppler effect elimination method for the ADBD to reduce time latency of fault diagnosis. To the authors’ knowledge, such a method has not been studied yet.

Motivated by the above analysis, an online fault diagnosis strategy called online Doppler effect elimination (ODEE) based on acoustic signal analysis technique is proposed in this paper for the ADBD. The analysis process of the proposed ODEE strategy is illustrated as follows: (1) the spatial and temporal parameters of the constructed kinematic model for the train movement is obtained in advance; (2) the proposed simplified unequal time interval strategy is taken and the distorted amplitude of the signal is demodulated as well; (3) the Doppler-free acoustic signal is restored and the characteristic frequency of the defective signal can be detected through the envelop spectrum of the restored Doppler-free signal. The procedure of the proposed ODEE strategy can be achieved online, which means the time consumption is less than that of the traditional offline strategy. Both simulation study and real train defective bearing signal process demonstrate that a remarkable performance is achieved by using the proposed ODEE method in the restoration of the Doppler distortion signal. Ultimately, the ODEE is implemented in a designed embedded system in bearing fault diagnosis and condition detection. The effectiveness of the proposed method is further verified. In summary, compared to the off-line strategy, the proposed ODEE strategy has the following properties: (1) the simplified unequal time interval strategy can be applied for online sampling to eliminate the Doppler effect; (2) The algorithms are designed for online running, e.g., the online envelope strategy; (3) A low-cost, low-powered, and high-efficiency embedded system is designed for the ODEE method, so the proposed ODEE technique is of low resource consumption and high efficiency when comparing to the traditional offline methods.

The rest of the paper is outlined as follows: Section 2 presents the principle of the proposed ODEE method. The simulation verification of the ODEE method is presented in Section 3. Afterwards, experimental verification of the ODEE method in the self-designed embedded system and discussions are presented in Section 4. Finally, conclusions are drawn in Section 5.

## 2. The Principle of the Proposed ODEE Method

### 2.1. Doppler Effect

**Figure 1:**
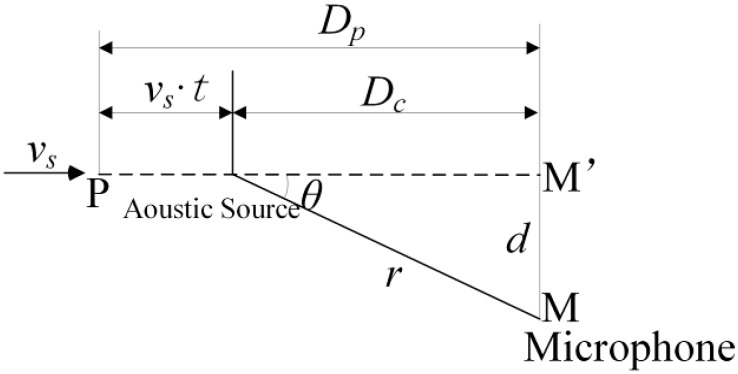
The basic kinematic model of a single acoustic source movement.

In the ADBD system, the microphones used to acquire the bearing acoustic signals from the passing train are placed perpendicular to the railway track by the wayside. Thus, a basic kinematic model for the train movement is constructed in detail in Figure 1. The single acoustic source is moving along a straight line while the microphone is not along the trail, which generates the nonlinear change of the distance between the acoustic source and the microphone. Therefore, the received acoustic signal contains the complicated nonlinear Doppler effect.

As illustrated in Figure 1, the single acoustic source is moving along a straight line at the velocity of *v_s_* from left to right, and the microphone is set at the point M, which is at a vertical distance of *d* to the moving orientation of the acoustic source. *r* donates the distance between the current position of the moving source and the microphone. Distance *D_P_* is measured from the initial position P to the position M’, which is the projection of the microphone on the motion trail. Thus, the relationship between the emission and reception frequency is presented as follows:

(1) $$–f_{\text{r}}=\left(\frac{c}{c-v_{s}\cos\theta \left(t\right)}\right)f_{\text{e}}$$

where *c* stands for the sound propagation velocity in air. *θ*(*t*) denotes the angle between the acoustic source moving orientation and the vector from the current position of the moving acoustic source to the microphone. *f_e_* stands for the emission frequency and *f_r_* represents the reception frequency.

Ultimately, the Doppler effect analysis and the constructed basic kinematic model are valuable for the ODEE method analysis, which allows the derivation procedure of the strategy to be easily understood.

### 2.2. The ODEE Principle

As shown in Figure 1, it is obvious that there exists time delay *dt* between the emission moment *t*_e_[*i*] and the reception moment *t*_r_[*i*], and *dt* stands for the sound propagation time in the air according to kinematic model. While the distance *r*_e_[*i*] between the microphone and the current position of the acoustic source at *t*_e_[*i*] can be obtained as:

(2) $$r_{\text{e}}\left[i\right]=\sqrt{d^{2}+{\left(D_{p}-v_{s}\cdot t_{\text{e}}\left[i\right]\right)}^{2}}$$

Thus, once the sound propagation speed *c* is regarded as a constant parameter, the relationship between *t*_e_[*i*] and *t*_r_[*i*] can be shown as:

(3) $$t_{\text{r}}\left[i\right]=t_{\text{e}}\left[i\right]+\sqrt{d^{2}+{\left(D_{p}-v_{s}\cdot t_{\text{e}}\left[i\right]\right)}^{2}}/c$$

It is observable that the variation of the distance *r*_e_ is nonlinear. Thus, if the emission moment {*t*_e_[*i*], *i* = 1,2,3,…,*N*} is selected as equal time intervals, unequal time intervals sampling strategy should be taken to obtain the corresponding amplitude of the reception moment.

In consideration of the complexity and difficulty to realize the real-time unequal time interval sampling, a simplified strategy is proposed. In according with the Nyquist-Shannon sampling theorem [26], for a given sampling rate *f*, prefect reconstruction can be guaranteed possible for a band limit *B* ≤ *f*/2, where *B* stands for the highest frequency of the origin signal. In the following study, the scheduled sampling frequency is defined as the appropriate one with which perfect restoration of the Doppler distorted signal can be achieved with traditional offline strategies, and it usually guaranteed to be more than four times of the highest wanted characteristic frequency of the origin signal. Thus, for the simplified strategy, a higher frequency *frs* that equals to *k* × *fs* (*k* > 2) is used to sample the Doppler distortion signal, where *fs* stands for the scheduled sampling frequency of the distorted acoustic source and *k* represents the frequency amplification factor that is taken for actual sampling process. Once the scheduled sampling frequency *fs* is determined, the corresponding emission moment vector {*t*_e_[*i*] = (*i* − 1)/*fs*, *i*=1,2,3,…,*N*} and the reception moment vector {*t*_r_[*i*], *i* = 1,2,3,…,*N*} are calculated. In the simplified real-time unequal interval sampling strategy, *t*_r_ is substituted approximately by using the sampling points which are the closest ones to the corresponding theoretical values in the actual sampling moment vector {*t*_s_[*i*] = (*i* − 1)/*frs*, *i* = 1,2,3,…,*kN*}. Thus, the signal can be restored with the demodulated value of selected effective sampling points in this way.

Assuming that if the value of *k* is infinite, the discrete sampling process turns into continuous sampling procedure, which means the accurate *t*_r_ can be acquired and the Doppler effect would be eliminated completely. However, it is difficult to realize an infinite sampling frequency for application. The higher the sampling frequency is, the more continuous the data and the higher the accuracy are. For the appropriate value selection, the relationship is established between the chosen *k* and the correlation coefficients of the original signal and the restored signal. Once a reasonable value of *k* is set for the actual sampling process, {*t*_r_[*i*], *i* = 1,2,3,…,*N*} are substituted approximately by using the selected actual effective sampling points {*n*[*i*], *i* = 1,2,3,…,*N*}. The selected points are chosen based on the formula below:

(4) $$n\left[i\right]=\left\lfloor t_{\text{r}}\left[i\right]\times frs\right\rfloor$$

which can be also written as below:

(5) $$n\left[i\right]=\left\lfloor \left(t_{\text{e}}\left[i\right]+\sqrt{d^{2}+{\left(D_{p}-v_{s}\cdot t_{\text{e}}\left[i\right]\right)}^{2}}/c\right)\times frs\right\rfloor$$

where ⌊•⌋ stands for the rounding and *n*[*i*] represents the selected actual effective sampling point.

Thus, the values of the effective sampling points can be obtained as {*x*[*n*[*i*]], *i* = 1,2,3,…,*N*}.

In accordance with the Morse acoustic theory [27], the received Doppler distortion signal is an amplitude distortion signal because of the variational distance between the moving source and the microphone. Thus, the aforementioned *x*[*n*[*i*]] is not the actual amplitude of the original signal.

Thus, the amplitude modulation should be taken for the obtained *x*[*n*[*i*]] as amplitude information is essential to the restored signal for more accurate diagnosis.

It is assumed that the propagation medium is perfectly fluid, which means that the medium has no viscosity and the propagation process has no energy loss. Meanwhile, the single moving acoustic source with subsonic velocity *M* = *v/c* < 1 could be simplified as a monopole point acoustic source. In this situation, the formula that used to describe the acoustic pressure *p* can be derived with the kinematic relation and the equation of waveform, that is:

(6) $$p=\frac{q^{\prime }\left(t-r/c\right)}{4\pi r{\left(1-M\cos\theta \right)}^{2}}+\frac{q\left(t-r/c\right)\left(\cos\theta -M\right)v_{s}}{4\pi r^{2}{\left(1-M\cos\theta \right)}^{3}}$$

where *q*(*t* – *r/c*) stands for the total quality flow rate of the point acoustic source and *q'*(*t* – *r/c*) is the derivation of *q*(*t* − *r/c*). *M* stands for the Mach number. When *M* < 0.2 and far-field measurement method is taken, the second part that demonstrates the near field effect is of small quantity compared with the first part and it can be ignored. Thus, the approximation of the formula is written as:

(7) $$p=\frac{q^{\prime }\left(t-r/c\right)}{4\pi r{\left(1-M\cos\theta \right)}^{2}}$$

Hence, according to the acoustic pressure field distribution equation, the amplitude modulation factor *P*_A_ can be written as:

(8) $$P_{\text{A}}=\frac{d}{r\left(t\right){\left(1-M\cos\theta \left(t\right)\right)}^{2}}$$

Therefore, if *x*_p_(*i*) stands for the amplitude demodulation signal, then it can be calculated as:

(9) $$x_{\text{p}}\left[i\right]=x\left[n\left[i\right]\right]/P_{\text{A}}\left[i\right]$$

which can also be written as:

(10) $$x_{\text{p}}\left[i\right]=x\left[\left\lfloor \left(t_{\text{e}}\left[i\right]+\sqrt{d^{2}+{\left(D_{p}-v_{s}\cdot t_{\text{e}}\left[i\right]\right)}^{2}}/c\right)\times frs\right\rfloor \right]\times r_{e}\left[i\right]\times {\left(1-M\cos\theta \left[i\right]\right)}^{2}/d$$

Finally, the Doppler-free signal can be obtained according to the analysis aforementioned and the characteristic frequency of the restored signal can be extracted for further analysis.

### 2.3. The Procedure of the Proposed ODEE Method

**Figure 2:**
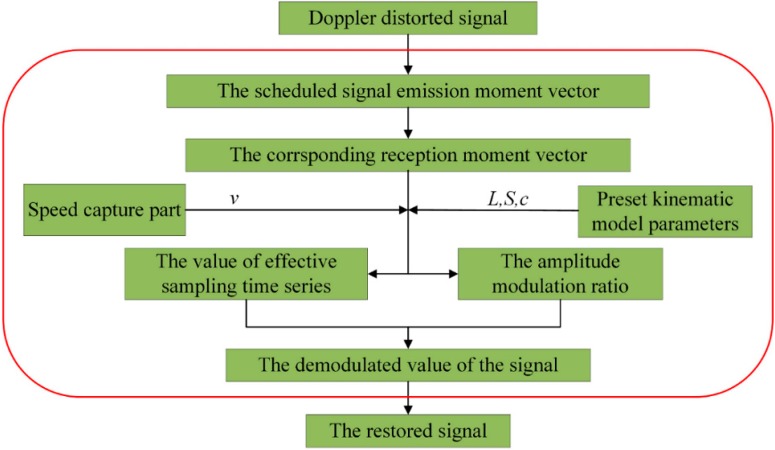
The flow chart of the proposed ODEE method.

As mentioned above, the signal is corrected in both frequency- and time-domain according to the proposed strategies. Ultimately, as illustrated in Figure 2, the steps of the ODEE method can be described as follows:

(1) The necessary parameters for the kinematic model are obtained based on the information acquired by the system in advance.
(2) The scheduled reception moment vector {*t*_r_[*i*], *i* = 1,2,3,…,*N*} is calculated on the basis of the derivation formula with the dynamic model analysis when the acoustic signal scheduled emission moment vector {*t*_e_[*i*], *i* = 1,2,3,…,*N*} is selected. The scheduled emission moment vector {*t*_e_[*i*]} is set to be {0, 1/*fs*, 2/*fs*, 3/*fs*, …, (*N* − 1)/*fs*} in general.
(3) Afterwards, the distorted signal is sampled by the system at the actual sampling frequency of *frs*, thus the actual sampling-time vector {*t*_s_[*i*], *i* = 1,2,3,…,*kN*} in the same duration can be denotes as {0, 1/*frs*, 2/*frs*, 3/*frs*, …, (*kN* − 1)/*frs*}.
(4) The calculated reception moment vector {*t*_r_[*i*], *i* = 1,2,3,…,*N*} is used to compute the effective sampling points{*n*[*i*], *i* = 1,2,3,…,*N*} which are the closest actual sampling points to *t*_r_. Then the values of the effective sampling series {*x*[*n*[*i*]], *i* = 1,2,3,…,*N*} are obtained and the amplitude modulation ratios {*P*_A_[*n*[*i*]], *i* = 1,2,3,…,*N*} are also figured out correspondingly.
(5) For the effective sampling series, the demodulation amplitude of the restored signal vector {*x*_p_[*i*], *i* = 1,2,3,…,*N*} is derived. Thus, the restored (Doppler-free) signal is obtained.

**Figure 3:**
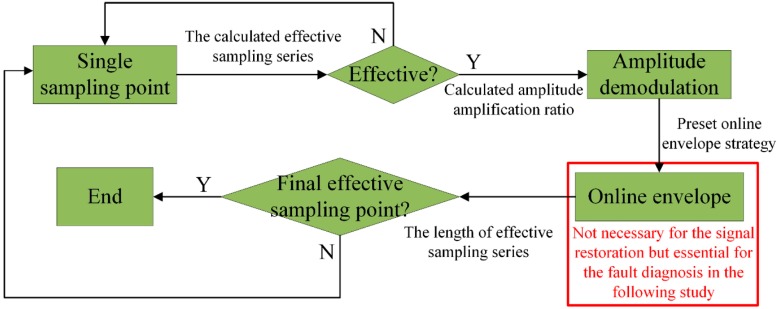
The process of each sampling point with the ODEE method.

Besides, to explicitly illustrate the proposed ODEE method, the process of each single sampling point is shown in Figure 3. The effective sampling series are calculated with the pre-measured kinematic model parameters and the scheduled sampling frequency in advance. Once the sampling procedure is started, whether the corresponding sampling point is effective or not is determined according to the calculated effective sampling series. If the sampling point is effective, the amplitude of the sampling point is demodulated with the corresponding amplitude modulation ratio and then the online envelope strategy of the effective point is taken for further analysis. Afterwards, the method comes to the next sampling point. In this way, the sampling point is processed one by one in sequence and the ODEE method is achieved online.

Additionally, an online envelope method is employed in this study for accelerating computation, which involves squaring the input signal and sending the obtained signal to a low pass filter. Generally, the obtained signal *x_p_*[*i*] with frequency modulation can be expressed as the product of a high frequency signal *x_p,h_* [*i*] and a low frequency signal *x_p,l_* [*i*], and it is written as

(11) $$x_{p}\left[i\right]=x_{p,h}\left[i\right]\times x_{p,l}\left[i\right]=A_{h}\cos\left[2\pi f_{h}i\right]\times \left[A_{l}\cos\left[2\pi f_{l}i\right]+c\right]$$

where *A_h_ and A_l_* represent the amplitude of the high frequency signal and the low frequency signal, respectively. *f_h_* stands for the corresponding high frequency and *f_l_* represents the low frequency. Besides, *c* is the DC bias voltage that makes the value of *A_i_*cos[2*πf_l_i*] + c greater than 0. Then a new signal *g*[*i*] which is twice as the square of the modulated signal is constructed as:

(12) $$\begin{array}{l} g\left[i\right]=2x_{p}^{2}\left[i\right]\\ \;=\;\left(\frac{1}{2}A_{l}^{2}A_{h}^{2}+c^{2}A_{h}^{2}\right)\cos\left[4\pi f_{h}i\right]\\ \;+\frac{1}{4}A_{l}^{2}A_{h}^{2}\left\{\cos\left[\left(4\pi f_{h}-4\pi f_{l}\right)i\right]+\cos\left[\left(4\pi f_{h}+4\pi f_{l}\right)i\right]\right\}\\ \;+cA_{l}A_{h}^{2}\left\{\cos\left[\left(4\pi f_{h}-2\pi f_{l}\right)i\right]+\cos\left[\left(4\pi f_{h}+2\pi f_{l}\right)i\right]\right\}\\ \;+\frac{1}{2}A_{l}^{2}A_{h}^{2}\left\{1+\cos\left[4\pi f_{l}i\right]\right\}+2cA_{l}A_{h}^{2}\cos\left[2\pi f_{l}i\right]+c^{2}A_{h}^{2} \end{array}$$

fIn the modulated signal, the high frequency *f_h_* is usually much higher than the low frequency *f_l_*. Therefore, if the constructed signal *g*[*i*] is sent to a low pass filter with a cut-off frequency lower than (2*f_h_* − 2*f_l_*), the signal components would be eliminated when the corresponding frequencies are higher than the selected cut-off frequency. Thus, the acquired signal *p*[*i*] after being filtered by the low pass filter can be expressed as:

(13) $$\begin{array}{l} p\left[i\right]=low\;pass\;filter\;(g\left[i\right])\\ \;=\frac{1}{2}A_{l}^{2}A_{h}^{2}\left\{1+\cos\left[4\pi f_{l}i\right]\right\}+2cA_{l}A_{h}^{2}\cos\left[2\pi f_{l}i\right]+c^{2}A_{h}^{2}\\ \;=A_{l}^{2}A_{h}^{2}\cos^{2}\left[2\pi f_{l}i\right]+2cA_{l}A_{h}^{2}\cos\left[2\pi f_{l}i\right]+c^{2}A_{h}^{2}\\ \;=A_{h}^{2}{\left(A_{l}\cos\left[2\pi f_{l}i\right]+c\right)}^{2} \end{array}$$

Hence, the envelope *x_e_* [*i*] of the modulated signal is finally obtained according to the square root of the filtered signal and it is shown as

(14) $$x_{e}\left[i\right]=sqrt\left(p\left[i\right]\right)=A_{h}A_{l}\cos\left[2\pi f_{l}i\right]+cA_{h}$$

In this study, IIR filter is selected as the low pass filter, and the filtered output *p*[*i*] can be expressed as the formula below:

(15) $$p[i]=-\sum_{k=1}^{M}a_{k}p[i-k]+\sum_{k=0}^{N}b_{k}g[i-k]$$

where the values of *M* and *N* are depended on the order and type of the selected filter. Once the cut-off frequency is also selected, the corresponding filter coefficients *a_k_ and b_k_* could be calculated. In this way, the low pass filter is achieved online in the algorithm for further analysis.

**Figure 4:**
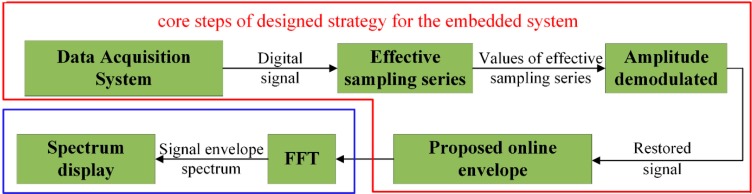
The flow chart of the algorithm for the designed embedded system.

Considering the flow chart of the proposed algorithm, the ODEE method can be implemented in the the low-cost, low-powered, and high-efficiency embedded system for better processing the signal online, and the flow chart of the algorithm for designed embedded system is shown in Figure 4. The obtained acoustic signal via the data acquisition system is first demodulated according to the pre-calculated effective sampling series with the proposed simplified unequal time interval strategy. At the same time, the amplitudes of the signal are restored via the amplification ratios computed in advance. Then, the online envelope of restored signal is calculated in the embedded system. Thus, the final results could be obtained by analyzing the power spectrum of the online envelope signal.

**Figure 5:**
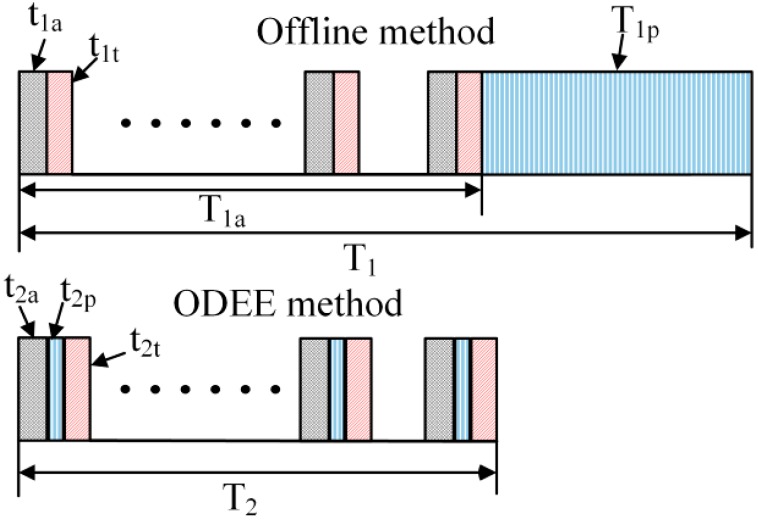
Time consumption comparison between the traditional method and the proposed ODEE method.

In the following, the ODEE method is compared with the traditional method in [23] to highlight its advantage. As shown in details in Figure 5, T_1_ stands for the total time consumption of the traditional offline strategy and T_2_ represents that of the proposed ODEE method. t_1a_ and t_2a_ represent the acquisition time for each sampling point in the traditional and the ODEE methods, respectively. Thus, it can be obtained that t_1a_ equals to t_2a_. Besides, T_1a_ is the total acquisition time for all the sampling points. t_1t_ = t_2t_ is the transmission time consumption for one sampled point and T_1p_ is the total time consumption of data processing procedure for the Doppler effect elimination through a traditional offline strategy. t_2p_ is the time consumed for the actual process of sampling at each point by using the ODEE method shown in Figure 3. It is obvious that T_2_ is approximately equal to T_1a_, which means that the unavoidable time latency T_1p_ in the traditional offline strategy could be eliminated by using the ODEE method. Therefore, the computation complexity of the ODEE method is lower than that of the traditional method. As time consumption is of vital importance, thus the proposed ODEE strategy is meaningful in online fault diagnosis of train bearings.

## 3. Simulation Verification of the ODEE Method

Subsequently, the effectiveness of the proposed ODEE method is verified according to the simulation analysis. Considering the aforementioned schematic diagram in detail in Figure 1, the relevant kinematic parameters are set as listed in Table 1. Besides, the actual sampling frequency *frs* is set as 50 kHz and the frequency amplification factor is set as 5. The original signal can be composed as follows:

(16) $$x_{o}=\sum_{i=1}^{5}q_{i}\cos(2\pi f_{i}t)+noise(t)$$

Therefore, on the basis of the derived formula, the simulative Doppler distortion fault signal corrupted by the stochastic noise can be obtained as:

(17) $$x_{d}=\sum_{i\text{=}1}^{5}\frac{2\pi q_{i}f_{i}\cos(2\pi f_{i}(t-(R/c)))}{4\pi R{\left(1-M\cos\theta \right)}^{2}}+noise(t)$$

where *f*_1_~*f*_5_ denote the five different frequency components in the simulated signal. With the purpose of verifying the effectiveness of proposed ODEE method in both high- and low-frequency, the frequencies are selected as listed in Table 2. *f*_3_ is adjacent to *f*_4_ to evaluate the effectiveness of correcting the Doppler distortion signal with frequency band confusion. Furthermore, the parameters of 2π*q_i_ f_i_/*4π is set to be 1 for simplicity. *noise*(*t*) denotes an additive Gaussian white noise, and the signal-to-noise ratio (SNR) of the signal is set as 10 dB.

**Table 1:** Parameters of the simulation Doppler distorted signal.

Parameter	*c*	*L*	*v*	*S*
Value	340 m/s	2 m	30 m/s	10 m

**Table 2:** Parameters of the selected characteristic frequency.

Parameter	*f*_1_	*f*_2_	*f*_3_	*f*_4_	*f*_5_
Value	200 Hz	800 Hz	1500 Hz	1550 Hz	2800 Hz

**Figure 6:**
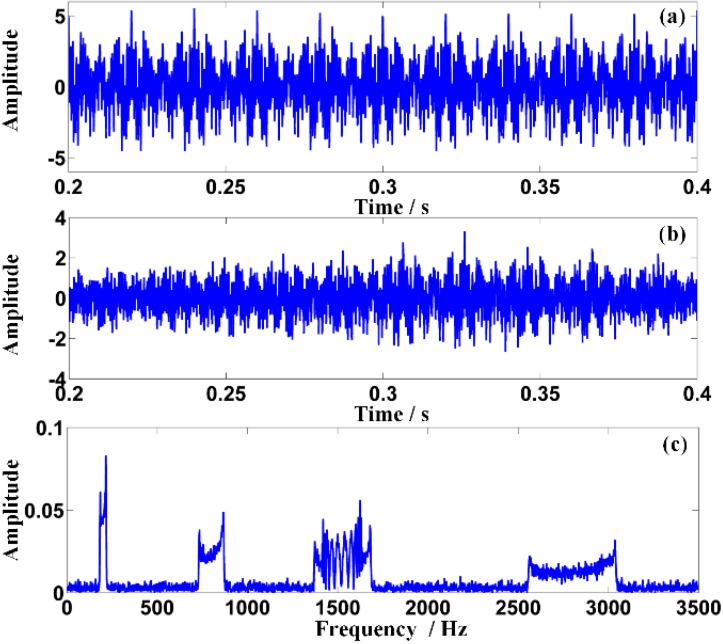
The Doppler effect of the simulated signal: (**a**) the waveform of the origin signal with SNR of 10 dB; (**b**) the waveform of the Doppler distorted signal; (**c**) the frequency spectrum of the Doppler distorted signal.

The waveforms of the origin signal and the distorted signal are shown in Figure 6a,b, respectively. The amplitude modulation can be observed clearly in Figure 6b. The frequency spectrum of the Doppler distorted signal shown in Figure 6c demonstrates the frequency shift and the frequency band extension. Besides, the higher the characteristic frequency is, the larger the frequency band extension is. Moreover, serious frequency band confusion appears between the scheduled adjacent characteristic frequencies *f*_3_ and *f*_4_. Therefore, the detected frequencies of the signal are not clear enough for recognition.

**Figure 7:**
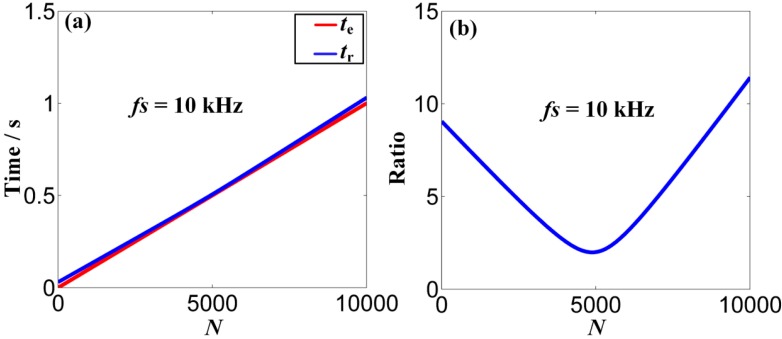
(**a**) Comparison between the scheduled emission time vector and the scheduled reception time vector with the scheduled frequency *fs* of 10 kHz; (**b**) the amplitude modulation ratio of the effective sampling series with the scheduled frequency *fs* of 10 kHz.

**Figure 8:**
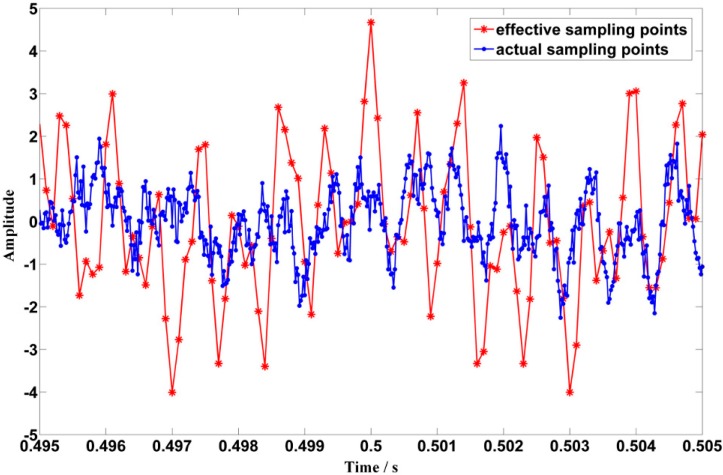
Comparison between the actual sampling series and the demodulated effective sampling series when the actual sampling frequency is 50 kHz.

**Figure 9:**
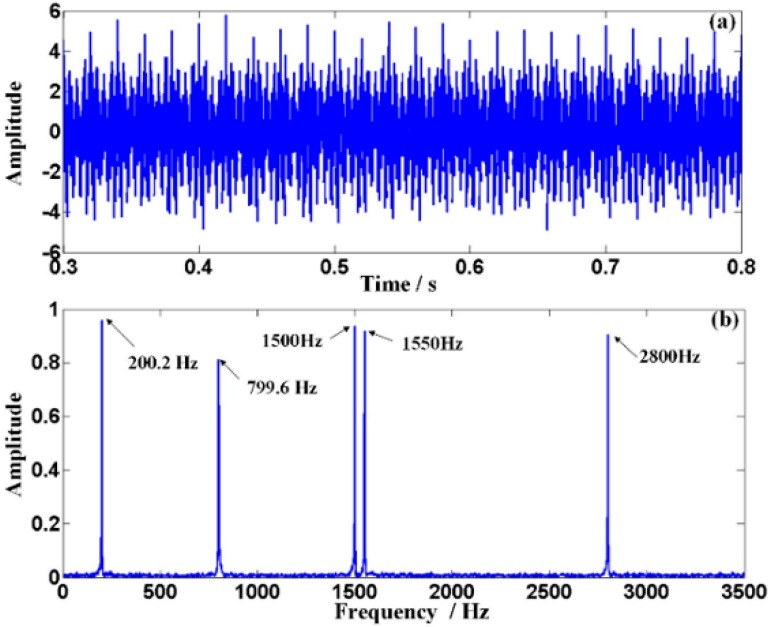
(**a**) The waveform of the restored signal via the ODEE method; (**b**) the spectrum of the restored signal.

To tackle the problem aforementioned and realize the proposed ODEE method, the proposed strategy is used to process the simulated Doppler distortion signal to validate the effectiveness. Firstly, a reasonable *fs* that equals to *frs/k* is selected. Thus, *t*_r_ is obtained according to the selected *fs* and the pre-obtained parameters of the kinematic model. Meanwhile, comparison between the selected *t*_e_ and calculated *t*_r_ is shown in Figure 7a. Afterwards, as the approximation relationship between the actual sampling moment series and the theoretical reception moment series mentioned above is established, the values of effective sampling time series are correspondingly obtained to reveal the frequency domain of the Doppler distorted signal. At the same time, with the corresponding amplitude demodulation ratios shown in Figure 7b, the amplitude distortions are demodulated. Afterwards, a comparison between the actual sampling series and the demodulated effective sampling series is conducted, which is employed to explicitly illustrate the processing procedure of the proposed strategy. The result is shown in detail in Figure 8. Finally, the Doppler effect elimination of the distorted signal is realized successfully and the result is shown in Figure 9, where the waveform and the spectrum of the restored signal are shown in Figure 9a,b, respectively. It is obvious that the frequency shift, the frequency band extension and confusion are all eliminated, and a satisfactory restoration of the Doppler distortion signal is achieved.

**Figure 10:**
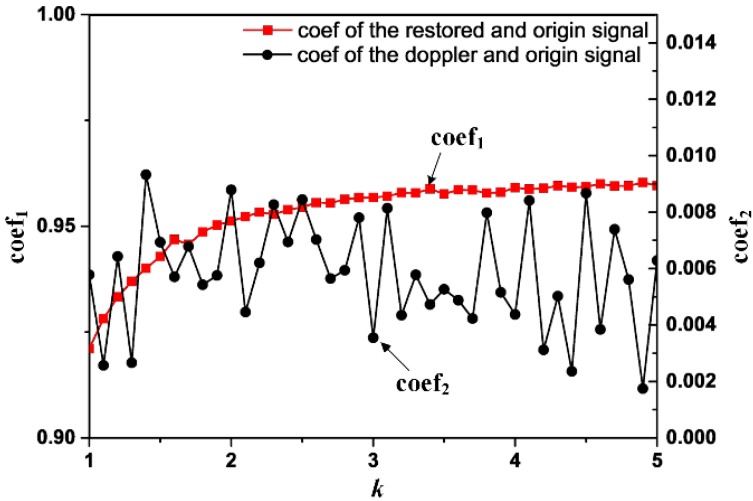
The relationships between *k* and coef_1_, coef_2_, respectively.

Moreover, correlation analysis is carried out between the origin signal and the restored signal to validate the effectiveness of the strategy proposed, where the correlative relationship between the selected *k* and *η_x_*_(*n*),*y*(*n*)_ of the origin signal and the restored signal is constructed. With the obtained analysis result and removal result of the Doppler distortion signal, it is convenient to select a reasonable *k* for the proposed processing procedure. As the stochastic noise would reduce the effectiveness of the analysis procedure significantly, in the following experiments, the SNR of the chosen signal *x_o_* is selected to be 10 dB. For each *k*, once the Doppler effect of the distorted signal is eliminated with the ODEE method mentioned above, the corresponding correlation coefficients can be calculated. The desired result of the correlative relationship is shown in Figure 10. The correlation coefficient of the origin signal and the obtained restored signal (coef_1_) increases with the growth of *k* and tends to be stabilized. While the correlation coefficient of the origin signal and the Doppler distortion signal (coef_2_) is of random distribution. Besides, coef_1_ is much higher than coef_2_, which verifies the effectiveness of the proposed ODEE method. Moreover, when *k* comes around 4.3, the correlation coefficient between the origin signal and the restored signal comes to 0.9596, which indicates that the restored signal is quite similar to the origin signal. The comparison result of the simulation verification implies the fairly good performance of the constructed ODEE method.

Besides, the implementation of the proposed ODEE method would become difficult to realize with the growth of *k*. Considering both accuracy and facilitation implementation, the value of *k* is chosen to be 5 during the simulation verification, which is high enough for the restoration procedure according to the correlation analysis. The results shown in details in Figure 9 also verified that the appropriate value of *k* has been chosen. Meanwhile, the same value of *k* has been selected in the following experimental verification procedure.

## 4. Experimental Verification of the ODEE Method in Embedded System

### 4.1. Experimental Setups for Signal Acquisition

As far as concerned, when there exists defect on the train bearing, periodic impulses will appear in the train bearing acoustic signal with the balls passing over the defect. Hence, the type of the bearing defect can be determined if the frequency of the periodic impulses generated by the defective bearing is obtained [28,29]. Besides, the defective characteristic frequency *f*_O_ of the bearing with outer-race defect can be calculated by the formula as follows:

(18) $$f_{\text{O}}=\frac{Z}{2}(1-\frac{d}{D})f_{\text{r}}$$

where *Z* stands for the number of rollers, *d, D* represents the diameter of roller and pitch diameter, respectively. *f*_r_ stands for the rotating speed. Similarly, the defective characteristic frequency *f*_I_ of the bearing with inner-race defect can be calculated by the equation as:

(19) $$f_{\text{I}}=\frac{Z}{2}(1+\frac{d}{D})f_{\text{r}}$$

Therefore, once the parameters of the used bearing and the rotating speed are given, the characteristic frequency of the defective bearing is determined. The type of train bearing defect can be classified according to the comparison.

After the effectiveness of the ODEE method has been verified in the simulation analysis, the proposed strategy is about to be employed in the embedded system to eliminate the Doppler effect of a real defective train bearing signal for further verification of the applicability and performance.

First of all, to make the designed system repeatable, the Doppler distortion signal should be obtained with the experimental setups for signal acquisition with Doppler effect described in the previous work [22,23]. The specifications of the tested bearing are listed in Table 3. Table 4 presents the parameters of the experiment to acquire the static signal without Doppler effect, including the radial direction load of the tested bearing, the rotation speed of the motor, and the sampling frequency. Thus, the defective characteristic frequencies *f*_O_ and *f*_I_ are calculated to be 138.7 Hz and 194.9 Hz, respectively. Besides, the necessary parameters of the dynamic experiment to obtain the Doppler distorted signal are shown in detail in Table 5.

**Table 3:** Specifications of the experiment bearing with the type of NJ(P)3226.

Pitch Diameter (*D*)	Roller Diameter(*d*)	Rollers Number(*Z*)
190 mm	32 mm	14

**Table 4:** Parameters of the first experiment.

Parameter	Radial Direction Load	Rotation Speed of the Motor	Sampling Frequency
Value	3 tons	1430 rpm	50 kHz

**Table 5:** Actual parameters of the Second constructed experiment.

Parameter	*c*	*L*	*v*	*S*
Value	340 m/s	2 m	30 m/s	6 m

Once the defective characteristic frequency of the acquired Doppler distortion signal is revealed, the type of train bearing defect can be determined via comparison with the aforementioned theoretical values. In the following analysis, the acquired Doppler distorted signal is used as the input acoustic signal of the designed embedded system to verify the effectiveness of the proposed ODEE method.

### 4.2. Experimental Setups of the Proposed Embedded System

**Figure 11:**
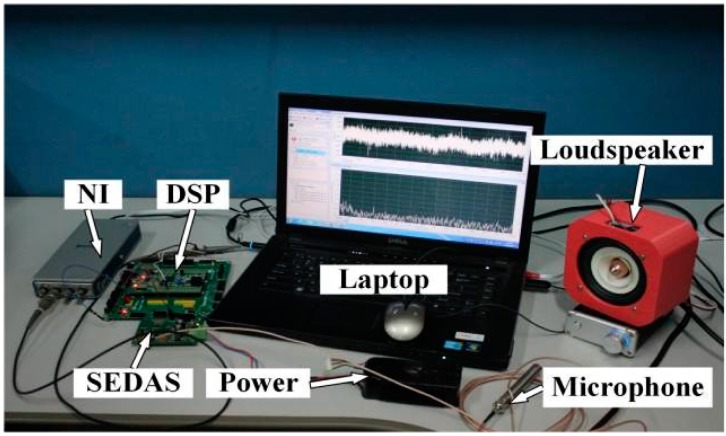
Experimental setups to validate the effectiveness of the ODEE method in the embedded system.

**Figure 12:**
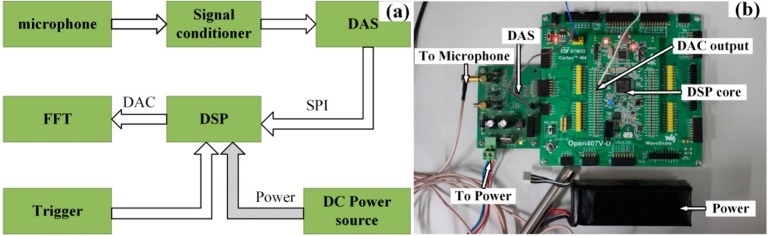
(**a**) The module diagram of the designed embedded system; (**b**) the hardware details of the embedded system.

As mentioned above, the input acoustic signal of the proposed embedded system are obtained according the designed experimental setups aforementioned. To realize the ODEE strategy by trackside for the train bearing fault diagnosis and condition monitoring, experiments are conducted to collect and process the Doppler distortion acoustic signals via the constructed embedded system. As shown in Figure 11, a loudspeaker is used to play the acquired actual Doppler distorted signals. A microphone is employed as the acoustic sensor for the signal acquisition of the embedded system. To accomplish the ODEE strategy, an embedded system composed by a self-designed embedded data acquisition system (SEDAS) and a digital signal processing (DSP) system is designed. The restored signal is processed via the system and the output can be observed on the laptop via the NI DAS to verify the effectiveness of the ODEE method. The module diagram of the designed hardware system is shown in Figure 12a, where the arrows represent the data flow direction. As a matter of fact, the constructed system is a signal reconstruction analyzer and modulator-demodulator that can realize the online Doppler effect elimination strategy.

The details of the designed hardware system are shown in Figure 12b. The maximal sampling rate of the SEDAS that is based on an analog-to-digital converter MAX1300 (Maxim Inc. Roanoke, VA, USA) is 115 ksps. The signal conditioner is composed of an operational amplifier MAX9632 (Maxim Inc. Roanoke, VA, USA) and the power supply for microphone is provided by an LM334 (STMicroelectronics Inc. Geneva, Switzerland). First of all, STM32F407VGT6 (STMicroelectronics Inc. Geneva, Switzerland) is based on the high-performance ARM Cortex-M4 platform. Besides, the floating-point processing unit (FPU) of the Cortex-M4 core supports all ARM single-precision data processing instructions and data types, which is essential for the restoration procedure of the proposed ODEE method such as the online envelope procedure, analog to digital transform. Additionally, it is a reduced instruction set computing (RISC) processor with a full set of DSP instructions to make the programming procedure easier. The designed application security can be enhanced by the memory protection unit (MPU) as well. Meanwhile, the incorporated high-speed embedded memories and the standard and advanced communication interfaces make it adaptive to complex situations. Furthermore, with the help of its DAC, the output can be transformed into analog signal and transmitted to other equipment for further analysis. The maximal frequency of the processor unit is up to 168 MHz, 210 DIPS. Moreover, the price is fairly low and the power consumption control is excellent. Thus, STM32F407VGT6 is chosen as the DSP core of the designed embedded system. With the help of the digital-to-analog converter embedded in the DSP, the restored Doppler-free signal can be collected with NI DAS and transmitted to the laptop that connected to NI DAS for further analysis.

Besides, a satisfactory restoration of the Doppler distortion signal could be achieved when the value of *k* is selected to be 5 according to the aforementioned simulation verification. Meanwhile, the complexity of implementation would be appropriate as well. Hence, the value of *k* has been selected to be 5 for the following experimental verification.

### 4.3. Case Study: Bearing with Outer-Race Defects

**Figure 13:**
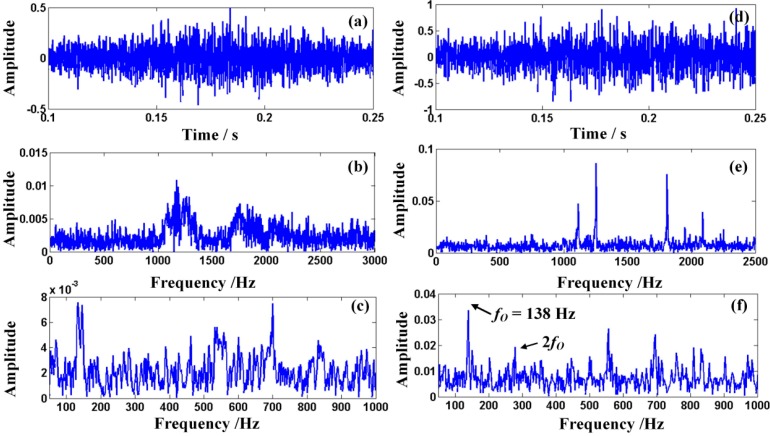
(**a**–**c**) The waveform, the spectrum and the envelope spectrum of the Doppler distorted signal emitted by the fault bearing with a outer-race defect; (**d**–**f**) the waveform, the spectrum and the envelope spectrum of the restored signal.

After obtaining the Doppler signal of the bearing with the outer-race defect by using the aforementioned experimental setups, the waveform and the power spectrum of the acquired Doppler distortion signal are described in detail in Figure 13a,b, respectively, and the envelope spectrum of the Doppler-shifted signal is drawn in Figure 13c. It is obvious that in the frequency domain there exists the frequency shift and the frequency band extension because of the Doppler distortion. Obvious amplitude modulation can also be found in Figure 13a. The characteristic frequency cannot be easily read from the current analysis. Hence, the defect cannot be easily detected from the envelope spectrum of the Doppler distorted signal. Afterwards, the distorted fault signal is first processed via the ODEE method on the laptop and the results are shown in detail in Figure 13d–f. The waveform of the restored signal is shown in Figure 13d, where the demodulated amplitude of the Doppler-free signal is displayed. The frequency spectrum and the envelope spectrum are drawn in Figure 13e,f, respectively. From Figure 13f, the characteristic frequency of the restored signal and its harmonic can be obtained. The characteristic frequency can be detected as 138 Hz, which is rather close to the theoretical value *f*_O_ calculated with the given parameters. From the comparison between the theoretical value and the calculated value, the proposed ODEE method correctly reveals the characteristic frequency of the defective bearing and the result validates the effectiveness of the proposed ODEE method running on the laptop.

**Figure 14:**
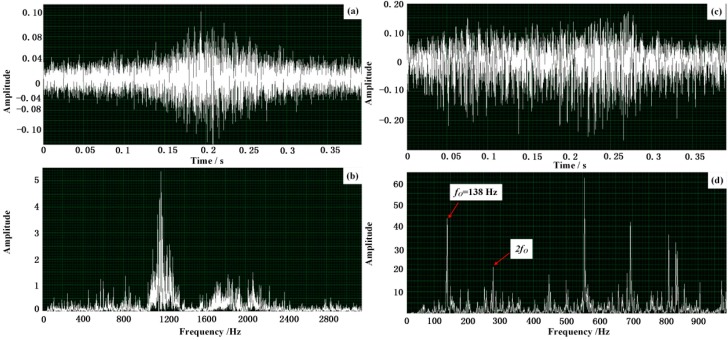
Effectiveness verification via the designed embedded system: (**a**,**b**) the waveform and the spectrum of the input Doppler distorted signal of the defective train bearing with a defect on the outer-race; (**c**,**d**) the waveform and the spectrum of the output restored envelope signal via the designed embedded system.

Subsequently, the acquired Doppler acoustic signal of the bearing with outer-race defect is taken in the designed system to validate the effectiveness of the algorithm implementation and hardware design. Firstly, the acoustic signal of the fault bearing with outer-race defect acquired by the aforementioned experiments is used as the input signal of the embedded system and its waveform and spectrum are displayed in Figure 14a,b, respectively. It is clear that the amplitude of the obtained signal is modulated according to Figure 14a and the frequency shift and frequency band extension can be observed according to Figure 14b. After the processing of the designed embedded system, the waveform and the frequency spectrum of the output restored envelope signal are drawn in Figure 14c,d, respectively, where the frequency shift and the frequency band extension shown in Figure 14b are successfully eliminated and the amplitude modulation shown in Figure 14a is demodulated. That implies the frequency domain of the acquired acoustic signal is restored and correctly reveals the feature frequency of the defective train bearing. The feature frequency of the defective train bearing can be clearly observed as 138 Hz, which is approximately equal to the calculated characteristic frequency *f*_O_ aforementioned. The effectiveness of the designed hardware embedded system has been verified.

### 4.4. Case Study: Bearing with Inner-Race Defect

**Figure 15:**
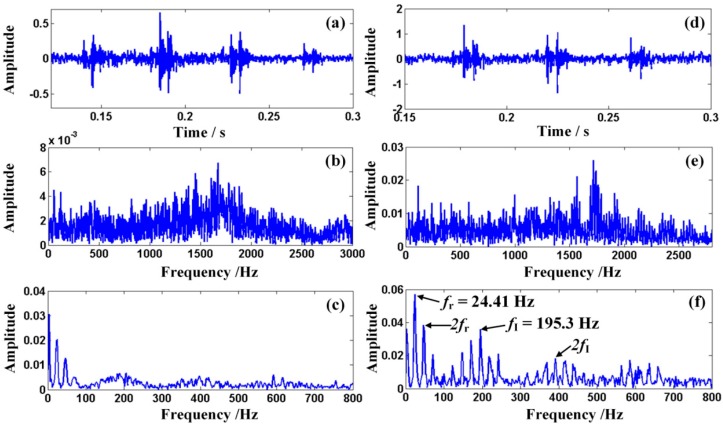
(**a**–**c**) The waveform, the spectrum and the envelope of the Doppler distorted signal emitted by the fault bearing with inner-race defect; (**d**–**f**) the waveform, the spectrum and the envelope spectrum of the restored signal.

Subsequently, similarly to procedure of bearing with the outer-race defect, the obtained Doppler signal of the bearing with inner-race defect is analyzed on the laptop as well. The waveform of the measured signal is shown in detail in Figure 15a, where obvious amplitude modulation can be observed. The frequency spectrum is shown in Figure 15b and the envelope spectrum is drawn in Figure 15c, where serious frequency shift and frequency band extension are observable. Hence, it is not easy to detect the type of bearing fault from the envelope of the obtained signal directly. The results of the analysis via the ODEE method are shown in detail in Figure 15d–f, where the demodulated amplitude, the frequency spectrum and the envelope spectrum of the Doppler-free signal are observable. The characteristic frequency of the restored signal can be detected as 195.3 Hz. It indicates a rather similar result to the theoretical value. Thus, it displays the characteristic frequency of the defective bearing signal correctly and the type of the fault bearing can be distinguished, which verifies the effectiveness of the ODEE method running on the laptop as well.

**Figure 16:**
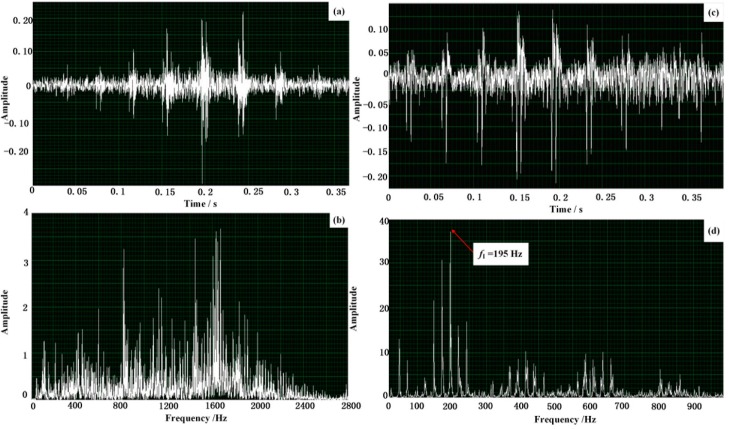
Effectiveness verification via the designed embedded system: (**a**,**b**) the waveform and the spectrum of the input Doppler distorted signal of the defective train bearing with a defect on the inner-race; (**c**,**d**) the waveform and the spectrum of the output restored envelope signal via the designed embedded system.

Subsequently, the input signal of the embedded system turns into the corresponding signal of the fault bearing with inner-race defect to verify the effectiveness of the designed embedded system. The waveform and spectrum of the Doppler distorted signal are displayed in Figure 16a,b, respectively. It is observable that the amplitude of the obtained signal is modulated and the frequency shift and frequency band extension take place in the frequency domain. Afterwards, the output of the constructed embedded system is shown in Figure 16c,d, which display the waveform and the spectrum of the output restored envelope signal, respectively. It is obvious that the frequency domain of the distorted signal has been restored and the feature frequency can be observed as 195 Hz, which is rather close to the aforementioned theoretical value *f*_I_ calculated with the given parameters. Thus, the characteristic frequency of the fault train bearing is displayed correctly, which again demonstrates the effectiveness of the constructed embedded system.

As a result, the above experiments verify the effectiveness of the designed hardware system in train bearing fault diagnosis and condition detection. The results show a potential application in analyzing the acoustic signal of the train bearings for fault diagnosis and condition detection by the trackside.

Besides, the proposed hardware system including the DSP, the DAS and the DC power is a low-cost implementation that costs less than $70. The current consumption of the designed system is 120 mA at 22 V DC, which means that the proposed hardware system is a low-powered system. In summary, the designed platform for real-time data acquisition and the Doppler effect elimination procedure is a low-cost, low-powered, and high-efficiency embedded system.

### 4.5. Discussions

The following presents some discussions for the proposed ODEE strategy.

(1) As known in this paper, the speed is regarded as a constant to simplify the dynamical model. In practice, the speed of the moving acoustic source may vary during the motion. In this regard, a more accurate instantaneous speed needs to be obtained for accurate diagnosis results. In further study, a more accurate dynamic model including the accelerated speed would be considered to improve the performance of the ODEE method.
(2) In this paper, a simplified unequal time interval sampling strategy is taken for the proposed ODEE method, which would bring in approximation error. An actual unequal time interval sampling strategy will be taken and implemented in the new embedded system for a more accurate diagnosis result.
(3) In the paper, the chosen speed is fairly high for a train bearing monitoring system. It might not be practical to listen to the train at this speed due to aerodynamic forces. In our further study, more reliable choices on the speed would be considered for application.

## 5. Conclusions

In the paper, to tackle the problem of Doppler distortion and implement the online Doppler effect elimination in the train bearing fault diagnosis and condition monitoring, a simple and effective method called ODEE is proposed. The Doppler effect including the frequency shift, frequency band expansion and amplitude modulation can be eliminated real-time, and both the simulation study and the real train bearing fault diagnosis proved that a satisfactory performance is achieved by the proposed ODEE method in removing the Doppler effect of the acoustic signal and diagnosing the bearing fault type. The ODEE strategy is carried out according to the following steps: the essential parameters are acquired in advance. Then, the simplified unequal time interval strategy is used to restore the Doppler distortion signal. The amplitude of the signal is demodulated as well. Hence, the restored Doppler-free signal is obtained online. The characteristic frequency of the acoustic signal can be acquired and the type of train bearing defect is identified accordingly. The real train bearing fault diagnosis shows that the constructed embedded system based on the proposed ODEE method can detect different conditions of the bearing for fault diagnosis and condition monitoring. Ultimately, the proposed method is implemented in an embedded system and the details of the constructed hardware system are presented. The effectiveness of the designed embedded system is validated with two different experiments in the end, which shows potential application for online train bearing fault diagnosis and condition monitoring by the trackside.
